# The Experience of Embodiment Scale: An examination of its psychometric properties in women from the Republic of Cyprus

**DOI:** 10.1371/journal.pone.0303268

**Published:** 2024-05-20

**Authors:** Viren Swami, Christophe Maïano, Marios Argyrides, Elly Anastasiades

**Affiliations:** 1 School of Psychology, Sport, and Sensory Sciences, Anglia Ruskin University, Cambridge, United Kingdom; 2 Centre for Psychological Medicine, Perdana University, Kuala Lumpur, Malaysia; 3 Cyberpsychology Laboratory and Department of Psychoeducation and Psychology, Université du Québec en Outaouais, Saint-Jérôme, Canada; 4 Department of Psychology, Neapolis University, Pafos, Cyprus; The World Islamic Sciences and Education University, JORDAN

## Abstract

The Experience of Embodiment Scale (EES) is a recently developed instrument that assesses experiences of living in the body. Here, we prepared a novel Greek translation of the EES and examined its psychometric properties. We initially prepared a Greek translation of the EES using a 5-step procedure recommended for test adaptation studies. Next, in a cross-sectional study, we asked a sample of 933 women from Cyprus to complete the Greek EES, alongside additional, previously validated measures assessing body appreciation, psychological well-being (self-esteem, life satisfaction), eating restriction, perfectionism, and internalisation of appearance ideals. Our analyses showed that EES factorial models based on confirmatory factor analysis (CFA) roundly had poor fit. Conversely, models based on exploratory structural equation modelling (ESEM)–which accounts for the fact that EES items cross-load across factors–had adequate fit to the data. Additionally, we found that both higher-order and bifactor-ESEM models that controlled for the uniqueness of negatively worded items had adequate fit. The bifactor-ESEM model had the best fit of all the models tested, and was invariant across ethnicity (Greeks and Greek-Cypriots) and was unaffected by differential item functioning based on age and body mass index. Additionally, construct validity of the final, optimal model was adequate, especially for its G-factor, as indicated by significant associations with additional constructs in expected directions. These results suggest that a bifactor-ESEM model of the Greek EES has adequate psychometric properties. Our work highlights important psychometric issues relating to the manner in which the EES should (or could) be conceptualised and modelled, which should be considered more fully in future work.

## Introduction

In its broadest sense, *embodiment* is a philosophical construct that refers to the “perceptual experience of engagement of the body in the world” [1] (p. 175), or the ways in which individuals live in and experience the world through their bodies. Although the construct of embodiment is central to understandings of body image, it had been relatively infrequently studied until the important work of Piran and colleagues [2–6], which sought to better understand the nature and antecedents of experiences of embodiment. Through extensive qualitative and cross-sectional research anchored in the experiences of North American girls and women, Piran [2, 3] developed the multidimensional *experience of embodiment* construct, which places embodiment on a continuum ranging from positive to negative experiences. Further, the experience of embodiment was conceptualised as being shaped by the quality of experiences along five dimensions, namely *body connection and comfort*, *agency and functionality*, *attuned self-care*, *inhabiting the body as a subjective site*, and *experience of expression of bodily desires*.

Based on this body of work, Piran and colleagues [7] went on to develop the Experience of Embodiment Scale (EES), the first instrument to specifically assess experiences of embodiment. To do so, they developed a pool of 48 novel items, of which 14 were excluded based on initial response patterns with data from sample of Canadian women (*N* = 92). Next, data from a new sample of Canadian women (*N* = 412) were subjected to exploratory factor analysis (EFA), which resulted in the extraction of six factors: Positive Body Connection and Comfort (PBCC; 6 items), Body Unencumbered Adjustment (BUA; 8 items), Agency and Functionality (AF; 7 items), Experience and Expression of Sexual Desire (EESD; 4 items), Attuned Self-Care (ASC; 6 items), and Resisting Objectification (RO; 3 items). Additionally, bifactor analysis suggested that it was possible to extract a stable general (G) factor and six specific (S) factors, with all but three items (#7, 18, and 19) loading onto the G-factor [7].

In a third study with women from the United States (*N* = 343), Piran et al. [7] tested three competing confirmatory factor analytic (CFA) models to determine the factor structure of the EES: a unidimensional model of EES scores, a higher-order model with six lower-order factors, and a bifactor model with a G-factor and six S-factors [7]. While the unidimensional model had poor fit, both the higher-order and bifactor-CFA models had adequate fit. Estimating the residual covariance of two items (#1 and 2) improved fit of the higher-order model, with all six lower-order factors adequately loading onto the higher-order factor. In contrast, seven items had weak loadings on the G-factor in the bifactor model and this, alongside the correspondence of the higher-order model with earlier qualitative research [2], led Piran and colleagues [7] to select the higher-order model as their preferred conceptualisation of the EES. Apart from the RO subscale (McDonald’s ω = .58), subscale scores on this model had adequate composite reliability (McDonald’s ω ≥ .78) and, across studies, the EES had adequate patterns of convergent and discriminant validity.

Given that these findings were largely anchored in the experiences of North American girls and women, it is unclear to what extent the 6-factor model of the EES will be recoverable in other national contexts. Indeed, to date, only one other study has examined the psychometric properties of the EES outside of North America [8]. Based on the results of an EFA using a novel Swedish translation of the EES with Swedish women (*N* ~ 295), Kling and colleagues [8] extracted a 6-factor model, with three factors (EESD, RO, and BCC) mirroring those reported by Piran and colleagues [7] and three remaining factors showing some deviation from the original model (e.g., all PBCC items and several BUA items formed a single factor). In Swedish men (*N* ~ 234), a 5-factor solution was preferred, with the AF and EESD factors notably being combined. In both cases, Kling and colleagues [8] also reported that it was possible to extract a higher-order model.

### Issues to consider

In view of these findings, the higher-order, 6-factor model of EES appears to be preferred by scholars, with studies utilising this model in analyses [9]. However, before this model is accepted as the optimal configuration of EES scores, particularly in cross-national research, further research on the psychometric properties of the EES in diverse national contexts is needed. In so doing, several inter-related issues should be considered.

#### Cross-loading items

The first of these issues relates to how cross-loading items should be dealt with. While there is no consensus in the literature concerning a minimum value above which a factor pattern coefficient is considered salient, even cross-loadings as low as .10 may sometimes exert salient effects on factor modelling [10]. This is particularly important because cross-loadings were common and substantive in both the original development study [7], as well as in the test adaptation work in Sweden [8]. For instance, examination of the item-factor loadings in the EFA reported by Piran and colleagues [7] shows that 11 of the 34 EES items cross-loaded on more than one factor at ≥ .25 and a majority (21 items) cross-loaded at ≥ .20. Likewise, although Kling et al. [8] did not report a full factor loading matrix, they noted that nine items had cross-loadings greater than .25.

This, in turn, has implications for how scholars should cross-validate the EES in test adaptation studies. Specifically, CFA may have lower utility as a method of cross-validation because CFA only allows items to load on to their respective hypothesised latent factor, while forcing cross-loadings to be zero [11–13]; that is, CFA assumes “pure” factors where these factors only load onto their *a priori* latent factors. However, given that a majority of EES items show at least some overlap across conceptually related facets, failure to account for cross-loadings will likely lead to biased estimates of factor correlations and associations between these factors and other variables [12, 14, 15], as well as at least some model misspecification [16]. Even apparently well-fitting CFA models–such as Piran and colleagues’ [7] 6-factor model–could hide these misspecifications given their ability to absorb unmodelled cross-loadings through an inflation of factor correlations, without letting them impact model fit, e.g., [13, 17].

Instead of relying on CFA, or indeed an EFA-to-CFA approach, an alternative method for assessing factorial validity where cross-loadings are assumed to be substantive is exploratory structural equation modelling (ESEM) [12, 17–19]. ESEM was specifically designed to integrate the best elements of both EFA and CFA, including the relaxation of the zero cross-loadings requirement of CFA (a feature typically limited to EFA), while also allowing researchers to obtain goodness-of-fit statistics, residual correlations, standard error estimates, tests of measurement invariance, and tests of associations between latent constructs (i.e., features typically limited to CFA). Moreover, through its “target” rotation method (i.e., where cross-loadings are “targeted” to be close to zero while all main loadings are freely estimated) [12, 17], ESEM allows for a theory-driven approach that can be used in a confirmatory manner. As a result, ESEM provides a different approach to address the aforementioned limitations of CFA for the assessment of factorial validity [17].

#### Higher-order modelling

There also remains scope to re-examine the utility of both higher-order and bifactor modelling of the EES. In their work, Piran and colleagues [7] in fact found that a 6-factor bifactor-CFA (henceforth B-CFA) model had superior fit to a 6-factor high-order CFA model (henceforth HO-CFA), although the former was de-valued on theoretical grounds (i.e., some items, including all RO items, did not load on the G-factor). While both bifactor and higher-order models postulate the co-existence of a general factor with specific factors, a unique feature of bifactor models is in their assumption that general factors have direct (rather than indirect) effects on the indicators (items). As such, all items are loaded on a specific factor and one general factor, with the variance of indicators partitioned into three sources, namely the specific factor, the general factor, and measurement error. In contrast, in higher-order models, associations between the indicators and the higher-order factor are indirect and associations between the indicators and the unique part of the higher-order factor are also mediated by the lower-order factors.

However, such an assumption in higher-order models is often viewed as empirically implausible and problematic [20, 21]. Indeed, emerging consensus suggests that bifactor models should be preferred over higher-order models unless there are strong conceptual and theoretical justifications for the latter [22]. With regards to the EES, Piran and colleagues’ [7] suggestion that the specific factors in their HO-CFA model can be conceptualised as subscale scores representing domains of the experience of embodiment is not in and of itself an adequate justification for favouring a higher-order model over a bifactor model. In fact, we suggest that it is in fact the bifactor model that provides greater theoretical and empirical plausibility [13]. Before any firm conclusions can be drawn, however, it should be noted that bifactor models can be constructed using both CFA and ESEM (henceforth B-ESEM). As such, and following on from the discussion above, there is a need to assess whether a B-ESEM model presents optimal fit for EES data.

#### Negatively worded items

A third issue that requires further consideration is the inclusion of negatively worded items. Notably, all items on the BUA factor were negatively worded and four of the six EES factors included both positively and negatively worded items. Although negatively worded items can sometimes be useful for minimising acquiescence, affirmation, or agreement biases, they also require greater cognitive effort when responding [23]. This, in turn, often leads to methods effects that result in spurious covariances among items. Such method effects can be viewed as “noise” variance that should be controlled in analyses or modelled as correlated uniqueness (CU) among the indicators [24]. Previous studies, however, have not controlled for negatively worded items in the EES, which may have resulted in biased parameter estimates.

#### Differential item functioning

Finally, much more can also be done to better understand whether respondent characteristics affect the probability of endorsing a given item on the EES (i.e., whether EES items are affected by differential item functioning or DIF). Two characteristics that are worthy of investigation in this regard are age and body mass index (BMI). In terms of the former, embodiment itself is a lived process that is likely to be affected by the physical state of one’s corporeal body, which in turn means that experiences of embodiment may be affected by age [25]. In terms of the latter, it is possible that navigating societies that objectify women based on BMI may affect experiences of embodiment. In Swedish women, for instance, Kling and colleagues [8] reported a negative association between EES scores and women’s BMI. In light of these issues, a fuller understanding of the role of age and BMI on item responses to the EES would be useful.

### The present study

In the present study, we sought to examine–with the above issues in mind–the psychometric properties of a novel Greek translation of the EES in a sample of women from Cyprus (officially the Republic of Cyprus), an island country in the Mediterranean Sea. Briefly, after almost eight decades under British rule, the majority Greek Cypriot population of Cyprus began pursuing a policy of *énosis* (union with Greece) in the 1950s, while the minority Turkish Cypriot population advocated for a policy of *taksim* (the partition of Cyprus and the creation of a Turkish polity in the north). When the island ultimately achieved independence rather than *énosis* in 1960, some of those disappointed by the failure of the *énosis* movement revived a campaign that resulted, in 1974, in a *coup d’état* against the elected president. This action precipitated the Turkish invasion of Cyprus, which led to the present-day partition of the island into the Republic of Cyprus (which has *de jure* sovereignty over the entire island but effectively controls about 60% of the island in the south and west) and the Turkish Republic of Northern Cyprus (considered an illegal occupation by the international community).

Because our sample in the present study is culturally novel, a number of analytic strategies are available to us. For instance, recent test adaptation work has utilised an EFA-to-ESEM/CFA strategy [26], in which an initial EFA is followed up with comparisons of fit of HO-CFA, HO-ESEM, B-CFA, and B-ESEM models. While such a strategy would normally be useful in test adaptation work, it is also atheoretical in its attempt to identify the best-fitting model for a dataset. In contrast, one could also accept that there is a need to balance theory and empiricism, while emphasising conceptual clarity. In this view, we accept as a starting point that the EES measures six facets of the experience of embodiment that are theoretically plausible [7]. That is, we honour the underlying research that has facilitated the development of the EES [2–6] and accept that the 6-factor model of EES scores offers a theoretically plausible account of the experience of embodiment, irrespective of the cultural or national context.

However, we also suggest that the EES factors are unlikely to be “pure” measures of their respective facets, but rather that the EES taps six highly inter-correlated factors that show substantive overlap at the level of the items and lower-order facets. As such, an alternative analytic strategy would be to begin by comparing the utility of 6-factor CFA and ESEM models, with and without CU, of the EES. Assuming that one or more of these models has adequate fit in the Cypriot setting, it would provide empirical support for the theoretical propositions that underlay the development of the EES. Next, we also cross-validate the optimal model and consider multidimensional functioning (i.e., higher-order and bifactor models), which would provide the most rigorous test of the factorial validity of the EES to date. This analytic framework has been employed previously [27] and allows us to honour the theoretical foundations of the EES while ensuring that empirical considerations are not minimised [28].

Based on the discussion above, we hypothesised that a 6-factor B-ESEM that accounts for CU would provide optimal fit to the data. Beyond an assessment of factorial validity, we also sought to extend knowledge in several ways. First, we considered the extent to which the optimal model of EES scores would demonstrate measurement invariance across ethnicity, which would give an additional degree of confidence that the optimal model is robust. Second, we considered the extent to which EES items may function differently based on respondent age and BMI. Given that the EES was designed to be reflective of the experiences of adult women, irrespective of their age and BMI [7], we should not expect to see DIF based on these respondent characteristics. Finally, we also conducted an examination of the construct validity of the EES in Cypriot women based on the availability of instruments that have been validated for use in Greek-speaking populations. Specifically, based on previous findings [7, 8, 29], we assessed: (a) convergent validity (expectation of a positive and moderate-to-high correlation with body appreciation); (b) concurrent validity (expectation of positive and moderate associations with self-esteem and life satisfaction, and negative and moderate associations with internalisation of appearance ideals and eating restriction); and (c) discriminant validity (expectation of a weak or negligible relationship with perfectionism).

## Materials and methods

### Participants

The total sample consisted of 933 respondents who identified as women. Only women were recruited because the EES was originally developed for, and validated with, women [7]. Participants ranged in age from 18 to 69 years (*M* = 37.31, *SD* = 9.75) and in self-reported BMI from 15.57 to 53.78 kg/m^2^ (*M* = 25.14, *SD* = 5.57). Most of the sample (62.1%) of the sample identified as Greek and 37.9% identified as Greek Cypriot. Of the sample, 0.5% had completed secondary education, 17.6% had completed college, 42.6% had an undergraduate degree, and 39.3% had a postgraduate degree. No further demographic data were collected.

### Measures

#### Experience of embodiment

Participants were asked to complete a novel Greek translation of the 34-item EES [7], with items rated on a 5-point scale ranging from 1 (*strongly disagree*; Greek: *διαφωνώ απόλυτα*) to 5 (*strongly agree*; Greek: *συμφωνώ απόλυτα*). To prepare a Greek translation of the EES, we followed the 5-step procedure recommended by Beaton et al. [30]. Specifically, two translators–one informed, and one uninformed–first independently forward-translated the EES instructions, items, and response options from English to Greek. Next, the two translations were examined by a third, independent translator who resolved any discrepancies and produce a synthesised translation. Third, the synthesised translation was then back-translated by two translators naïve to the EES back into English. Fourth, the forward- and back- translations were compared by an expert committee comprising all the translators, as well as all authors of the present study, who resolved any minor inconsistencies between versions. In the fifth and final stage, the translated EES was pre-tested in a sample of 16 women who broadly matched the target sample. Participants in the pre-test study provided qualitative feedback regarding their level of understanding, as well as suggestions for improvements to enhance comprehension (based on open-ended questions). This feedback was returned to the committee, who agreed that no further revisions were necessary. The EES items in English and Greek are reported in the Appendix within the (S1 Appendix).

#### Body appreciation

Participants completed the 10-item from the Body Appreciation Scale-2 (BAS-2) [31]; Greek translation: [32]. Items were rated on a 5-point scale (1 = *never*, 5 = *always*). In the present study, McDonald’s ω for scores on this scale was .96 (95% CI = .95, .96).

#### Self-esteem

Self-esteem was assessed using the 10-item Rosenberg Self-Esteem Scale (RSES) [33]; Greek translation: [34]. Items were rated on a 4-point scale (1 = *strongly disagree*, 4 = *strongly agree*. In the present study, McDonald’s ω for RSES scores was .91 (95% CI = .90, .92).

#### Life satisfaction

Participants completed the 5-item Satisfaction with Life Scale (SLS) [35]; Greek translation: [36], which assesses one’s assessment of the quality of life on the basis of their own unique criteria. All items were rated on a 5-point scale ranging from 1 (*strongly disagree*) to 5 (*strongly agree*). In the present work, McDonald’s ω for scores on this scale was .90 (95% CI = .89, .91).

#### Eating restriction

To measure eating restriction, we used the 5-item Restraint subscale of the Eating Disorder Questionnaire (EDE-Q) [37]; Greek translation: [38]. All items were rated on a 7-point scale, ranging from 0 (*no days*) to 6 (*every day*). In the present work, McDonald’s ω for scores on this subscale was .85 (95% CI = .83, .86).

#### Perfectionism

To measure perfectionism, we used the High Standards subscale of the Almost Perfect Scale–Revised (APS-R) [39]; Greek translation: [40], a 7-item measure of adaptive perfectionism. All items were rated on a 7-point scale ranging from 1 (*strongly disagree*) to 7 (*strongly agree*). In the present work, McDonald’s ω for scores on this subscale was .86 (95% CI = .84, .87).

#### Internalisation of appearance ideals

Participants were asked to complete the Internalisation-General subscale of the Sociocultural Attitudes Toward Appearance Questionnaire-3 (SATAQ-3) [41]; Greek translation: [42], a 9-item measure of the degree to which individuals internalise general appearance ideals. All items were rated on a 5-point scale (1 = *strongly disagree*, 5 = *strongly agree*). In the present study, McDonald’s ω for scores on this subscale was .94 (95% CI = .93, .95).

#### Demographics

Participants were asked to provide their demographic details consisting of age, highest educational qualification, ethnicity, height, and weight. Height and weight data were used to compute BMI as kg/m^2^.

### Procedures

Ethics approval was obtained from the relevant departmental ethics committee (approval code: EEBK ΕΠ 2022.01.74), and permission to modify the scale was obtained from the owner of the original Experience of Embodiment Scale. Participants were recruited via a Google Forms link that was promoted through advertisements placed on social media platforms. Those who expressed an interest were first asked to complete a pre-screener to determine eligibility for the study. Inclusion criteria included 18 years of age or older, their preferred language being Greek, and being a citizen or resident of the Republic of Cyprus. Those who met the inclusion criteria provided digital informed written consent after being presented with additional information about the study, including that participation was anonymous, voluntary, and without remuneration. All participants then completed a survey consisting of all the measures described above presented in a counter-balanced order (to control for order effects), and four attention checks placed randomly throughout the questionnaire. Internet Protocol (IP) addresses were checked to ensure that no participant completed the survey more than once. All data were collected between August and November 2022.

### Statistical analyses

#### Data treatment

Data used in the present study are provided in S1 File. To examine the dimensionality of EES scores, our aim was to initially compare the utility of CFA and ESEM models, before cross-validating these results. To ensure adequate subsample sizes for both sets of analyses, the total dataset was first split using a computer-generated random seed, resulting in two split-half subsamples (*n* = 467 and 466, respectively). There were no significant differences between the two split-halves in terms of age and BMI, nor were there significant differences in the distribution of ethnic groups and educational qualifications (results available from the corresponding author). There were no missing data in our dataset.

#### First split-half subsample

*CFA and ESEM representations of the EES*. In the first split-half subsample, all analyses were conducted using Mplus 8.8’s [43] robust weighted least squares estimator with mean and variance adjusted statistics (WLSMV). In a first step, the *a priori* 6-factor model of the EES was estimated using CFA and ESEM. In the CFA model, EES ratings were respectively explained by six correlated latent factors without cross-loadings. In the ESEM solution, all cross-loadings were freely estimated using a confirmatory oblique target rotation procedure, allowing us to rely on an *a priori* specification of the main indicators of each factor and “targeting” all cross-loadings to be as close to zero as possible [14, 44]. Additionally, in order to control for the methodological artefact introduced by the negatively worded EES items [24, 45], CFA and ESEM models were also separately estimated with CU between the following items: #21 and 31 (from the Agency and Functionality factor); #28 and 30 (from the Experience and Expression of Sexual Desire factor); #15, 16, 18 and 23 (from the Attuned Self-Care factor); and #19 (from the Resisting Objectification factor). Composite reliability of EES latent factors was estimated using McDonald’s omega (ω) [46].

*Model fit*. Model fit was examined using the following fit indices [47–49]: the root mean square error of approximation (RMSEA) and its 90% CI (values ≤ .08 indicate acceptable fit; ≤ .06 indicates good fit), the Tucker-Lewis index (TLI; values ≥ .90 indicate acceptable fit and > .95 indicate good fit), and the comparative fit index (CFI; values ≥ .90 indicate acceptable fit and > .95 indicate good fit). However, as highlighted by Morin et al. [13, 17], goodness-of-fit assessment is insufficient to guide model selection when contrasting ESEM and CFA. Therefore, Morin et al. [13, 17] also recommended carefully examining parameter estimates (i.e., loadings, cross-loadings, latent correlations, composite reliability) from ESEM and CFA models. Observation of reduced factor correlations in the ESEM solution coupled with generally well-defined factors presents additional evidence for the superiority of the ESEM solution over a similarly fitting CFA solution [13, 17].

*Ethnic invariance*. The model providing the optimal representation of the data (CFA or ESEM, with and without CU) was then retained for tests of measurement invariance across ethnicity in the following sequence [50]: (i) configural invariance; (ii) weak invariance (loadings); (iii) strong invariance (thresholds); (iv) strict invariance (uniquenesses); (v) invariance of CU (if the model with CU is retained); (vi) invariance of the latent variances/covariances; and (vii) invariance of latent mean factors. Model comparisons (i.e., with each model contrasted to the previous one) relied on changes (Δ) in CFI, TLI, and RMSEA. Invariance was supported when ΔCFI and ΔTLI were ≤ .01, and ΔRMSEA was ≤.015 [51, 52].

*Differential item functioning and latent mean differences as a function of age and BMI*. In a third step, a hybrid multiple indicators multiple causes (MIMIC) multiple-group model [53–55] was used to examine: (a) differential item functioning (DIF), that is, direct associations between the predictors (age and BMI) and the EES item responses over-and-above the association between the predictors and the EES latent factors; (b) the associations between predictors (age and BMI) and EES latent factors; and (c) the equivalence of these associations across the two ethnic groups (Greek and Greek-Cypriot). These models were developed from the most invariant multiple-group model identified in the ethnic invariance test, to which the age and BMI were included. Specifically, hybrid MIMIC models were estimated in the following sequence [18, 19]: (a) null effects model (paths from the predictors to the EES latent factors and item responses were constrained to be zero); (b) saturated model (paths from the predictors to the EES item responses were freely estimated, while paths from the predictors to the EES latent factors were constrained to be zero); and (c) factors only model (paths from the predictors to the EES latent factors were freely estimated, while paths from the predictors to the EES item responses were constrained to be zero). To ease interpretations, age and BMI were standardised prior to analyses. A substantial improvement in model fit (ΔCFIs-ΔTLIs > .01 and ΔRMSEAs >. 015 [55], in the factors-only and saturated models relative to the null effects model provides support for an association between predictors and the EES item responses. Additionally, improvement in model fit for the saturated model relative to the factors-only model provides support for DIF. These models were studied with all associations freely estimated across the Greek and Greek-Cypriot subsamples. Then, the most appropriate model was retained and compared to an alternative model in which all associations were constrained to be equal across Greek and Greek-Cypriot respondents.

#### Second split-half subsample

*Higher-order and bifactor representations of the EES*. In the second split-half subsample, all analyses were conducted using Mplus 8.8’s [43] robust WLSMV. In a first step, the *a priori* 6-factor model of the EES was estimated using CFA and ESEM as described in 2.4.2.1. Higher-order representation (HO-CFA and HO-ESEM) of the EES was estimated using six *a priori* first-order factors (PBCC, BUA, AF, EESD, ASC, and RO) and one second-order factor (HO of experience of embodiment). The HO-ESEM was estimated using ESEM-within-CFA as recommended by Morin and Asparouhov [56]. Bifactor representation of the EES (B-CFA and B-ESEM) comprised one more factor than their CFA and ESEM counterparts. In these models, all factors were specified as orthogonal [13, 17] and all items had a main loading on both a global factor (G-factor of experience of embodiment) and on their six specific factors (S-factors: PBCC, BUA, AF, EESD, ASC and RO). Additionally, based on results obtained in the first split-half subsample, models were estimated with or without CU. Finally, McDonald’s ω [46] was used to estimate composite reliability of EES latent factors.

*Model fit*. Fit indices used to identify the optimal model were identical to those reported above. As highlighted by Morin et al. [13, 17] goodness-of-fit assessment is insufficient to guide model selection when contrasting CFA, ESEM, HO-CFA, HO-ESEM, B-CFA, and B-ESEM solutions. Instead, Morin et al. [13, 17] recommended a careful examination of parameter estimates (i.e., loadings, cross-loadings, latent correlations, composite reliability) from the various models. This comparison begins with a comparison of the CFA and ESEM models, where the observation of reduced factor correlations in the ESEM solution coupled with generally well-defined factors provides evidence in favour of the ESEM solution over a similarly fitting CFA solution [13, 17]. Next, the retained model should be contrasted to its higher-order and bifactor counterparts. In this second comparison, the observation of a well-defined G-factor coupled with at least a subset of well-defined S-factors supports the superiority of the bifactor solution over a similarly fitting first-order or higher-order solution [13, 17].

*Ethnic invariance*, *DIF*, *and latent mean differences as function of age and BMI*. These analyses were performed with the model providing the most optimal representation of the data (CFA, B-CFA, HO-CFA, ESEM, HO-ESEM, and B-ESEM), using the same strategies as those reported in Section 2.4.2.3.

*Construct validity*. Construct validity was examined in the total sample using a structural equation model (SEM) in which the EES factor structure was estimated based on model providing optimal representation of the data. In this model, the EES latent factors and the observed scores of convergent or concurent measures were all correlated. Correlations values ≤ .10 were considered weak, ~ .30 moderate, and ~ .50 strong [57].

## Results

### First split-half subsample

#### CFA and ESEM representations of the EES

Goodness-of-fit indices of all measurement models are reported in Table 1. The 6-factor CFA solution had poor fit to the data (TLI < .90 and RMSEA > .08), whereas the 6-factor CFA-CU reached acceptability for CFI and TLI (> .90), but not for RMSEA (> .08). Both ESEM models (with and without CU) showed substantial improvement in fit relative to their CFA counterparts (ESEM: ΔCFI = +.051, ΔTLI = +.039, ΔRMSEA = -.020; ESEM-CU: ΔCFI = +.045, ΔTLI = +.037, ΔRMSEA = -.020). Additionally, the 6-factor ESEM-CU solution had an improved level of fit (ΔCFI = +.008, ΔTLI = +.007, ΔRMSEA = -.004) relative to its non-CU counterpart. Although these results lend preliminary support to the ESEM-CU solution relative to the CFA-CU solution, we followed Morin et al.’s [13, 17] suggestions, turning our attention to the parameter estimates from these solutions.

**Table 1:** Goodness-of-fit statistics for the Experience of Embodiment Scale (EES).

Models	Sample	N°	Description	Wχ^2^	*df*	CFI	TLI	RMSEA	RMSEA 90% CI		CM	ΔWχ^2^	*df*	*p*	ΔCFI	ΔTLI	ΔRMSEA
									LB	UB							
CFA	First split-half	1–1	6-factor	2691.139*	512	.907	.898	.095	.092	.099	-	-	-	-	-	-	-
		1–2	6-factor-CU	2327.644*	476	.921	.907	.091	.088	.095	-	-	-	-	-	-	-
ESEM	First split-half	1–3	6-factor	1343.313*	372	.958	.937	.075	.070	.079	-	-	-	-	-	-	-
		1–4	6-factor-CU	1122.174*	336	.966	.944	.071	.066	.075	-	-	-	-	-	-	-
ESEM: MI across ethnicity	First split-half	2–1	Greek	622.972*	336	.968	.947	.070	.061	.079	-	-	-	-	-	-	-
		2–2	Greek-Cypriot	772.911*	336	.970	.949	.067	.060	.073	-	-	-	-	-	-	-
		2–3	Configural invariance	1373.743*	672	.970	.950	.067	.062	.072	-	-	-	-	-	-	-
		2–4	Weak invariance	1388.062*	840	.976	.969	.053	.048	.058	2–3	268.61	168	< .001	+.006	+.019	-.014
		2–5	Strong invariance	1424.995*	934	.979	.975	.047	.042	.052	2–4	104.25	94	.22	+.003	+.006	-.006
		2–6	Strict invariance	1450.222*	968	.979	.976	.046	.041	.051	2–5	58.67	34	.01	.000	+.001	-.001
		2–7	CU invariance	1476.100*	1004	.980	.977	.045	.040	.050	2–6	30.87	36	.71	+.001	+.001	-.001
		2–8	Variances-covariances invariance	1213.110*	1025	.992	.991	.028	.021	.034	2–7	26.48	21	.19	+.012	+.014	-.017
		2–9	Latent means invariance	1207.768*	1031	.992	.992	.027	.020	.033	2–8	3.82	6	.70	.000	+.001	-.001
DIF: Age and body mass-index	First split-half	3–1	MIMIC Null effects	1664.416*	1167	.977	.975	.043	.038	.047	-	-	-	-	-	-	-
		3–2	MIMIC Saturated	1259.350*	1031	.990	.987	.031	.024	.037	3–1	540.16	136	< .001	+.013	+.012	-.012
		3–3	MIMIC Factors only	1386.249*	1143	.989	.988	.030	.024	.036	3–1	204.98	24	< .001	+.012	+.013	-.013
		3–4	MIMIC Factors only (invariance)	1418.304*	1155	.988	.987	.031	.025	.037	3–3	28.75	12	.004	-.001	-.001	+.001
CFA	Second split-half	4–1	6-factor-CU	2216.366*	476	.913	.898	.089	.085	.092	-	-	-	-	-	-	-
HO-CFA		4–2	1 HO-factor and 6 first order factors—CU	2219.700*	485	.914	.900	.088	.084	.091	-	-	-	-	-	-	-
B-CFA		4–3	1 G-factor and 6 S-factors—CU	1818.882*	457	.932	.917	.080	.076	.084	-	-	-	-	-	-	-
ESEM		4–4	6-factor-CU	1068.605*	336	.964	.939	.068	.064	.073	-	-	-	-	-	-	-
HO-ESEM		4–5	1 HO-factor and 6 first order factors—CU	1008.804*	345	.967	.946	.064	.060	.069	-	-	-	-	-	-	-
B-ESEM		4–6	1 G-factor and 6 S-factors—CU	646.258*	308	.983	.969	.049	.043	.054	-	-	-	-	-	-	-
B-ESEM: MI across ethnicity	Second split-half	5–1	Greek	521.526*	308	.981	.966	.049	.042	.056	-	-	-	-	-	-	-
		5–2	Greek-Cypriot	456.254*	308	.984	.971	.052	.041	.061	-	-	-	-	-	-	-
		5–3	Configural invariance	1010.461*	616	.981	.966	.052	.047	.058	-	-	-	-	-	-	-
		5–4	Weak invariance	1201.271*	805	.981	.974	.046	.040	.051	5–3	330.82	189	< .001	.000	+.008	-.006
		5–5	Strong invariance	1293.938*	896	.981	.976	.044	.038	.049	5–4	134.57	91	.002	.000	+.002	-.002
		5–6	Strict invariance	1329.322*	930	.981	.977	.043	.038	.048	5–5	59.59	34	.004	.000	+.001	-.001
		5–7	CU invariance	1375.411*	966	.980	.977	.043	.037	.048	5–6	69.37	36	< .001	-.001	.000	.000
		5–8	Variances-covariances invariance	1148.714*	994	.993	.992	.026	.018	.032	5–7	33.13	28	.23	+.013	+.015	-.017
		5–9	Latent means invariance	1151.294*	1001	.993	.992	.025	.017	.032	5–8	7.07	7	.42	.000	.000	-.001
DIF: Age and body mass-index	Second split-half	6–1	MIMIC Null effects	1699.932*	1137	.969	.966	.046	.041	.051	-	-	-	-	-	-	-
		6–2	MIMIC Saturated	1186.353*	1001	.990	.987	.028	.021	.034	6–1	672.45	136	< .001	+.021	+.021	-.018
		6–3	MIMIC Factors only	1308.548*	1109	.989	.988	.028	.021	.034	6–1	280.29	28	< .001	+.020	+.022	-.018
		6–4	MIMIC Factors only (invariance)	1330.148*	1123	.989	.987	.028	.021	.034	6–3	21.75	14	.08	.000	-.001	.000

*Notes*. EES = Experience of Embodiment Scale; Wχ^2^ = robust weighed least square (WLSMV) chi-square; df = degrees of freedom; CFI = comparative fit index; TLI = Tucker-Lewis index; RMSEA = root mean square error of approximation; 90% CI = 90% confidence interval of the RMSEA; LB = lower bound; UB = upper bound; CM = comparison model; Δ = change from the previous model; ΔWχ^2^ = WLSMV chi square difference test (calculated with the Mplus DIFFTEST function); CFA = confirmatory factor analyses; CU = correlated uniqueness; ESEM = exploratory structural equation modeling; MI = measurement invariance; DIF = differential item functioning; MIMIC = multiple indicators multiple causes; HO-CFA = higher-order CFA; B-CFA = bifactor CFA; G-factor = global factor; S-factor = specific factor; HO-ESEM = higher-order ESEM; B-ESEM = bifactor ESEM

* p ≤ .01.

Parameter estimates for the 6-factor CFA-CU and 6-factor ESEM-CU solutions are reported in S1 and S2 Tables^1^ in S1 File. In the 6-factor CFA-CU solution, factor loadings were all reasonably high (PBCC: *M*_λ_ = .854; BUA: *M*_λ_ = .680; AF: *M*_λ_ = .798; EES: *M*_λ_ = .833; ASC: *M*_λ_ = .712; RO: *M*_λ_ = .637), except for Item #19 on the RO factor. These factors presented acceptable coefficients of composite reliability (ω = .705 to .943, *M*_ω_ = .869) and their latent correlations remained high (*r* = .354 to .867; *M*_*r*_ *=* .593).

In the 6-factor ESEM-CU solution, factor loadings were generally acceptable (PBCC: *M*_λ_ = .632; BUA: *M*_λ_ = .498; AF: *M*_λ_ = .718; EES: *M*_λ_ = .730; ASC: *M*_λ_ = .564; RO: *M*_λ_ = .608) and accompanied by reasonably small cross-loadings (*M*_λ_ = .107), with the exception of Items #3, 16, 19, 21, and 22. Item #3 from the BUA factor was more strongly associated with the PBCC factor; Item #16 from the ASC factor presented a similar pattern of associations with the BUA factor; Item #19 from the RO factor was more highly associated with the BUA factor; Item #21 from the AF scale presented a similar pattern of associations with the ASC factor; and Item #22 from the ASC factor presented a similar pattern of associations with the AF factor. The composite reliability coefficients of the six factors were adequate (ω = .718 to .923; *M*_ω_ = .847). In contrast to the 6-factor CFA-CU solution, the latent factor correlations between scales were substantially reduced (*r* = .221 to .558; *M*_*r*_ *=* .352), thus supporting their distinguishability (see S2 Table in S1 File).

#### Ethnic invariance

Goodness-of-fit statistics associated with the ESEM-CU solutions estimated separately in the Greek and Greek-Cypriot subsamples are presented in Table 1 (Models 2–1 and 2–2). These results revealed acceptable fit indices for all models (CFI and TLI > .90 or > .95; RMSEA ≤ .08). The goodness-of-fit statistics associated with the tests of measurement invariance conducted for the ESEM-CU (Models 2–3 to 2–9) provided support for complete measurement invariance (i.e., loadings, thresholds, uniqueness, CU, variances/covariances, and means) of the ESEM-CU model.

#### DIF as function of age and BMI

Table 1 presents the results from the MIMIC models. These models were estimated starting from the most invariant model of the ethnic invariance test (model 2–9: invariance of means). Results revealed a substantial improvement in model fit in the saturated (model 3–2) and factors-only models (model 3–3) relative to the null effects model (model 3–1). This suggests that the predictors (age and BMI) are associated with EES responses. Additionally, the factors-only model resulted in a similar level of model fit compared to the saturated model (ΔCFI = -.001, ΔTLI = +.001, ΔRMSEA = -.001), supporting a lack of DIF as a function of predictors. Finally, the last model (model 3–4), built from the retained factors-only model, showed that relations between the predictors and the latent factors could be considered as equivalent across Greek and Greek-Cypriot subsamples.

Table 2 presents results from the invariant factors-only model. First, these results showed that age significantly and positively predicted scores on the BUA, ASC, and RO factors. More specifically, older participants tended to present significantly higher scores on these subscales. Second, BMI significantly and negatively predicted scores on the PBCC, BUA, EESD, and ASC subscales. Thus, individuals with higher BMIs tended to present significantly lower values on these subscales.

**Table 2:** Relations between the EES latent factors and the predictors in the first and second split-half subsamples.

* *	First Split-Half Subsample (ESEM-CU)				Second Split-Half Subsample (B-ESEM-CU)			
			Subsample-specific standardised coefficients				Subsample-specific standardised coefficients	
	*b*	(SE)	*β* (Greek)	*β* (Greek-Cypriot)	*b*	(SE)	*β* (Greek)	*β* (Greek-Cypriot)
*Age *								
G-factor	*-*	*-*	*-*	*-*	.145	(.049)**	.130**	.129**
Positive Body Connection and Comfort	.074	(.049)	.069	.068	.113	(.059)	.112	.113
Body Unencumbered Adjustment	.125	(.056)*	.115*	.114*	.322	(.064)**	.307**	.306**
Agency and Functionality	.090	(.052)	.089	.089	-.001	(.054)	-.001	-.001
Experience and Expression of Sexual Desire	-.064	(.052)	-.063	-.063	-.144	(.056)*	-.142*	-.142*
Attuned Self-Care	.187	(.051)**	.180**	.179**	.096	(.053)	.093	.094
Resisting Objectification	.215	(.058)**	.210**	.210**	-.036	(.058)	-.034	-.034
*Body mass-index *								
G-factor	*-*	*-*	*-*	*-*	-.510	(.055)**	-.458**	-.454**
Positive Body Connection and Comfort	-.404	(.052)**	-.377**	-.376**	.001	(.061)	.001	.001
Body Unencumbered Adjustment	-.439	(.058)**	-.405**	-.402**	-.162	(.058)**	-.154**	-.153**
Agency and Functionality	.081	(.047)	.081	.081	.247	(.054)**	.240**	.240**
Experience and Expression of Sexual Desire	-.130	(.051)**	-.128**	-.129**	.065	(.056)	.064	.064
Attuned Self-Care	-.264	(.046)**	-.254**	-.252**	.196	(.069)**	.191**	.191**
Resisting Objectification	.042	(.055)	.041	.041	.330	(.065)**	.314**	.314**

*Notes*. EES = Experience of Embodiment Scale; ESEM = Exploratory Structural Equation Model; B-ESEM = bifactor ESEM; CU = correlated uniqueness; b = unstandardized regression coefficient taken from the factors-only models (3–4 and 6–4) invariant across samples; SE = standard error of the coefficient; β = sample-specific standardized regression coefficient (although some of the relations are invariant across samples, the standardised coefficients may still show some variation as a function of within-samples estimates of variability); G-factor = global factor

* p ≤ .05

** p ≤ .01.

### Second split-half subsample

#### Higher-order and bifactor representations of the EES

Goodness-of-fit indices of all measurement models are reported in Table 1. The 6-factor CFA-CU solution had poor fit to the data (TLI < .90 and RMSEA > .08). Although the B-CFA-CU model reached acceptability (CFI and TLI > .90; RMSEA = .08), the HO-CFA-CU reached acceptability for CFI and TLI (> .90), but not for RMSEA (> .08). In contrast, the ESEM-CU, HO-ESEM-CU, and B-ESEM-CU solutions resulted in an acceptable fit to the data and in a level of fit that was substantially improved relative to their CFA-CU (ΔCFI = +.051, ΔTLI = +.041, ΔRMSEA = -.021), HO-CFA-CU (ΔCFI = +.053, ΔTLI = +.046, ΔRMSEA = -.024), and B-CFA-CU counterparts (ΔCFI = +.051, ΔTLI = +.052, ΔRMSEA = -.031). The comparison of the first-order, higher-order, and bifactor ESEM solutions suggested the superiority of the B-ESEM-CU compared to the ESEM-CU (ΔCFI = +.019, ΔTLI = +.030, ΔRMSEA = -.019) and HO-ESEM-CU solutions (ΔCFI = +.016, ΔTLI = +.023, ΔRMSEA = -.015). However, despite the results seeming to favour the B-ESEM-CU solution, as recommended by Morin et al. [13, 17], the selection of the optimal solution should be based on a careful examination of the parameter estimates, composite reliability, and factor correlations. To this end, the CFA-CU and ESEM-CU solutions were first contrasted, and then the most optimal of these representations with its higher-order and bifactor counterparts.

The detailed parameter estimates from all solutions are reported in S3-S8 Tables^2^ in S1 File. In the CFA-CU solution, the factor loadings of the EES were satisfactory (|*λ*| = .308-.939, *M*_λ_ = .701) and associated with acceptable composite reliability coefficients (ω = .716 to .936, *M*_*ω*_ = .868). However, the factor correlations remained high (*r* = .563). In contrast, this factor correlation was substantially reduced in the ESEM solution (*r* = .334), suggesting that the slight level of overlap could in fact be explained by the presence of cross-loadings (*M*_|λ|_ = .108). Nevertheless, although most cross-loadings remain negligible, the results also suggest that (a) Item #3 from the BUA subscale was more strongly associated with the PBCC subscale; (b) Item #17 from the PBCC subscale presented a nearly similar pattern of associations with the ASC subscale; (c) Item #19 from the RO subscale was more highly associated with the BUA subscale; and (d) Item #22 from the ASC subscale presented a nearly similar pattern of associations with the AF subscale. Finally, the ESEM solutions also resulted in generally well-defined (|*λ*| = .197-.894, *M*_λ_ = .618) and reliable latent factors (ω = .671 to .906, *M*_*ω*_ = .841).

Based on these above results, the ESEM solution was favoured, and contrasted with the HO-ESEM-CU and B-ESEM-CU solutions. In the HO-ESEM-CU solution, the first-order factor loadings of the EES were generally acceptable (|*λ*| = .252-.901, *M*_λ_ = .618) and accompanied by reasonably small cross-loadings (*M*_λ_ = .107) with the exception of Items #3, 8,17, 19, and 22. The composite reliability coefficients of the six first-order factors were adequate (ω = .691 to .905, *M*_*ω*_ = .844). Additionally, the second-order factor was well-defined by five of the subscales of the EES (|γ| = .532-.761, *M*_γ_ = .631), but weakly defined by the RO scale (γ = .293) suggesting that this scale might tap into a different construct. The composite reliability coefficient of the second-order factor was adequate (ω = .754).

The B-ESEM-CU solution resulted in a well-defined G-factor (|*λ*| = .053-.853, *M*_λ_ = .584) reflecting participants’ experience of positive embodiment, except for one item (#19) that remained substantial in two S-factors (BUA and RO). Additionally, the composite reliability of the G-factor was excellent (ω = .971) and higher than of the HO-ESEM-CU solution (ω = .754). Moreover, results revealed that: (a) the PBCC was less well defined (|*λ*| = .024-.674, *M*_λ_ = .282, ω = .692) and this could be attributed to four items (Items #8, 9, 11, and 17) that essentially serve to define the G-factor; (b) the BUA (|*λ*| = .221-.446, *M*_λ_ = .359, ω = .720) and ASC (|*λ*| = .218-.539, *M*_λ_ = .380, ω = .693) S-factors remained reasonably well-defined, but with weaker loadings values than those of the G-factor; (c) the AF (|*λ*| = .402-.738, *M*_λ_ = .535, ω = .846) and EESD (|*λ*| = .533-.704, *M*_λ_ = .598, ω = .834) S-factors remained well-defined and with similar loadings values to those of the G-factor; and (d) the RO (|*λ*| = .340-.729, *M*_λ_ = .592, ω = .710) S-factor remained well-defined and with higher loadings values than those of the G-factor. Finally, most cross-loadings were reduced (except for Items #3, 19, 22, and 34) in the B-ESEM-CU solution (*M*_|λ|_ = .083) relative to the ESEM-CU and HO-CFA-CU (*M*_|λ|_ = .107) solutions. Therefore, the present results support the B-ESEM-CU representation of the data (i.e., B-ESEM-CU resulted in an improved level of fit to the data compared to all other solutions; the G-factor of experience embodiment is a well-defined and the EES S-factors are reasonably well-defined).

#### Ethnic invariance

Table 1 presents goodness-of-fit statistics associated with the B-ESEM-CU solutions estimated separately in the Greek and Greek-Cypriot subsamples (Models 5–1 and 5–2). These results revealed good fit indices for all models (CFI and TLI > .95; RMSEA ≤ .06). Additionally, the goodness-of-fit statistics associated with the tests of measurement invariance conducted for the B-ESEM-CU (Models 5–3 to 5–9) provided support for the complete measurement invariance (i.e., loadings, thresholds, uniqueness, CU, variances/covariances, and means) of the B-ESEM-CU solution.

#### DIF as function of age and BMI

Results from the MIMIC models are reported in Table 1. These models were estimated starting from the most invariant model of the ethnic invariance test (model 5–9: invariance of means). Results revealed a substantial improvement in model fit in the saturated (model 6–2) and factors-only models (model 6–3) relative to the null effects model (model 6–1). This supports the idea that the predictors (age and BMI) are associated with EES responses. Additionally, the factors-only model resulted in a similar level of model fit compared to the saturated model (ΔCFI = -.001, ΔTLI = +.001, ΔRMSEA = .000), supporting a lack of DIF as a function of predictors. Finally, the last model (model 6–4), built from the retained factors-only model, showed that relations between the predictors and the latent factors could be considered equivalent across subsamples.

Table 2 presents result from the invariant factors-only model. First, these results showed that age significantly and positively predicted the G-factor and the BUA S-factor of the EES. Additionally, age significantly and negatively predicted the EESD S-factor of the EES. More specifically, older participants tended to present significantly higher values on the G-factor and the BUA S-factor, and significantly lower values on the EESD S-factor. Second, BMI significantly and negatively predicted the G-factor and the BUA S-factor of the EES. Additionally, BMI significantly and positively predicted the AF, ASC and RO S-factors of the EES. Thus, individuals with higher BMIs tended to present significantly lower values on the G-factor and the BUA S-factor, and higher values on the AF, ASC and RO S-factors.

#### Construct validity

Goodness-of-fit statistics from the SEM including the EES latent factors (B-ESEM-CU model) and the other measures were acceptable: χ^2^(470) = 1570.347, CFI = .973, TLI = .955, RMSEA = .050 (90% CI = .047, .053). As illustrated in Table 3, the EES G-factor was significantly and (a) positively correlated with body appreciation, internalisation of appearance ideals, life satisfaction, perfectionism, and self-esteem; and (b) negatively correlated with eating restriction. Results for the PBCC and EESD S-factors showed significant and positive correlations with eating restriction. Additionally, results for the BUA S-factor also showed significant and (a) positive correlations with body appreciation, internalisation of appearance ideals, perfectionism and self-esteem; and (b) negative correlations with eating restriction and perfectionism. Moreover, results showed that the AF S-factor was significantly and positively correlated with all measures. In addition, the ASC S-factor was significantly and positively correlated with self-esteem, and negatively correlated with perfectionism. Finally, the RO S-factor was significantly and (a) positively correlated with body appreciation and internalisation of appearance ideals; and (b) negatively correlated with eating restriction, life satisfaction, and perfectionism.^3^

**Table 3:** Construct validity analyses from the bifactor exploratory structural equation modelling representation with correlated uniqueness of the EES in the overall sample.

	PBCC	BUA	AF	EESD	ASC	RO	G-Factor
	S-factor	S-factor	S-factor	S-factor	S-factor	S-factor	
Body appreciation	.047	.059**	.038*	.003	-.004	.040*	.885**
Eating restriction	.075*	-.283**	.160**	.116**	-.029	-.152**	-.347**
Internalisation of appearance ideals	.024	.304**	.065*	.003	-.009	.422**	.412**
Life satisfaction	-.010	-.036	.215**	-.002	.036	-.076*	.532**
Perfectionism	.049	-.175**	.337**	.033	-.154**	-.126**	.196**
Self-esteem	.024	.113**	.425**	-.007	.086**	-.042	.686**

*Notes*. EES = Experience of Embodiment Scale; PBCC = Positive Body Connection and Comfort; S-factor = specific factor; BUA = Body Unencumbered Adjustment; AF = Agency and Functionality; EESD = Experience and Expression of Sexual Desire; ASC = Attuned Self-Care; RO = Resisting Objectification; G-factor = global factor

* p ≤ .05

** p ≤ .01.

## Discussion

The present study assessed the factorial validity and psychometric properties of a novel Greek translation of the EES in women from Cyprus. In broad outline, our results showed that a 6-factor B-ESEM model of EES scores that controls for CU had optimal fit and, additionally, was superior in terms of fit compared to all other models that were tested. Importantly, our results also indicated that CFA-based models generally had poor fit and, relative to ESEM models, had inferior fit. Beyond factorial validity, we also found that the B-ESEM-CU model was invariant across ethnicity, was not impacted by differential item functioning on the basis of respondent age and BMI, and generally showed adequate convergent, concurrent, and discriminant validity. Below, we begin by discussing the poor fit of CFA-based models before turning our attention to the optimal model of the EES in our sample.

### CFA-based models had relatively poor fit

One key finding from the present study was that the 6-factor CFA model and the same model controlling for CU had poor fit to our data. Likewise, we also found that 6-factor CFA model that controlled for CU and that included a higher-order dimension (i.e., HO-CFA-CU) had poor fit, whereas the counterpart CFA model that included a bifactor only just about achieved adequate fit (i.e., B-CFA-CU). While scholars should avoid using fit indices as “golden rules” to judge model fit (e.g., [58]), these findings do not provide resounding support for CFA-based modelling of the Greek EES. Indeed, we might also note that the HO-CFA model tested by Piran and colleagues [7]) also had sub-par fit, which led the authors to introduce a correlation between Items #1 and 2 in order to achieve acceptable fit. However, such *post hoc* corrections are sub-optimal and may still hide misspecifications given the ability of CFA to absorb unmodelled cross-loadings through an inflation of factor correlations, without letting them impact model fit (e.g., [13, 17]).

In other words, the present findings are consistent with our suggestion that CFA-based models are problematic *vis-à-vis* the EES because they assume that each EES factor only loads onto their *a priori* latent factors and ignores the fact that EES items overlap across factors. Of course, one might be tempted to argue at this point that, given that the B-CFA-CU model had adequate fit, this model should be preferred over any other models precisely because subscale scores in these models can be viewed as representing domains of experience of embodiment [7]. However, we suggest that such an argument would be misplaced for three reasons. First, it ignores the fact that the EES items do overlap, and often quite substantively. Second, across CFA-based models, inter-factor correlations were moderate-to-high, suggesting a substantive degree of nomological overlap (i.e., the different “pure” factors are in fact measuring something common at a lower-order level). Third, and perhaps most importantly, CFA-based models consistently had relatively poorer fit compared to ESEM-based models.

### ESEM-based models consistently had good fit

Our results showed that the ESEM-based models consistently had adequate fit to our data and relatively superior fit compared to the CFA-based models. In the first split-half subsample, both ESEM models (with and without CU) showed superior fit compared to their CFA counterparts, whereas in the second split-half subsample the B-ESEM-CU model had excellent fit to the data and was the best-fitting model of EES scores overall. On this basis alone, researchers should be encouraged to consider the possibility that ESEM-based models may provide a more optimal model of EES scores, but there are additional reasons to favour this structure over others. First, given that EES items do overlap across factors–sometimes substantially–ESEM-based models would seem to offer a more realistic and plausible account of the EES factor structure. Second, compared to the CFA-based solutions, latent factor correlations between subscales in the ESEM models were substantially reduced (i.e., the ESEM-based models appear to have reduced artificially inflated inter-factor correlations compared to CFA-based models, which in return reduces the degree or multicollinearity; [59]). Indeed, the fact that factor correlations were attenuated in the ESEM would seem to suggest that any nomological overlap is in fact being caused by the presence of cross-loadings, which CFA-based models ignore.

One potential objection to the ESEM-based models of EES scores is that they are relatively complex and, given this complexity, samples must be sufficiently large to conduct analyses. While we do not necessarily disagree, we also suggest that complex multidimensional scoring methods will be necessary for the future development of the EES and theoretical understandings of the experience of embodiment. Indeed, given that different scoring methods (i.e., CFA versus ESEM) conceptualise slightly different components of the experience of embodiment, researchers need to be aware of these differences and how they affect our ability to answer specific research questions. Likewise, if scholars are hopeful of using the EES to understand experiences of embodiment across social identity groups (e.g., sexual orientation, gender identity, nationality, etc.), where measurement invariance is a prerequisite, then they will need a model of EES that is robust, theoretically plausible, and conceptually meaningful.

### Bifactor versus higher-order modelling

While the results from the second split-half subsample indicated that the B-ESEM-CU model had the best fit of the models tested, we also found that the HO-ESEM-CU had adequate fit. Given that both models achieved adequate fit, which should be preferred? We suggest there are several reasons to prefer the bifactor over the higher-order model. First, composite reliabilities of the G-factor in the bifactor model were substantively higher than the reliability of the counterpart higher-order EES factor. Second, cross-loadings were substantively reduced in the bifactor compared to the higher-order model. Finally, and similar to the findings of Piran and colleagues [7], the higher-order factor of EES scores was well-defined by only five of the six EES subscales (i.e., the RO subscale only weakly defined the higher-order construct, suggesting that this facet may tap a different construct when the experience of embodiment is modelled as a higher-order construct). In contrast, the S-factors were reasonably well-defined in the bifactor model and contributed to the G-factor, which represent the theoretical conceptualisation of the experience of embodiment.

Put differently, while the higher-order modelling of EES scores may be more convenient, this method may be less closely aligned with theoretical formulations of the experience of embodiment. Of course, one may suggest that, given that the RO factor was not theorised *a priori* [2], it is not a core facet of EES and could be discounted in future work. This is ultimately a theoretical discussion that goes beyond the present study, but our work suggests that modelling the EES using bifactor analysis would allow researchers to examine the role of overall embodiment (i.e., the G-factor), as well as the unique characteristics of all six facets of the experience of embodiment (i.e., the S-factors). It is also worth noting that the B-ESEM-CU model is not influenced by multicollinearity, as are the other multidimensional methods assessed here. Instead, in B-ESEM, all commonality between subscales is modelled through the G-factor and inter-factor correlations are constrained to zero.

### Other findings

A clearer example of the potential nuance of the bifactor model can be seen in the relationships between EES scores and additional variables included in the present study. While the G-factor was significantly correlated with all additional variables largely in the expected directions, associations with S-factors were less clear-cut, with the BUA and AF S-factors most closely mirroring our hypothesised expectations. In contrast, associations with the additional S-factors were often attenuated and sometimes non-significant, suggesting that the B-ESEM-CU model is able to separate what is common versus specific across experience of embodiment facets (and, therefore, may offer a theoretically consistent approach to modelling the construct of the experience of embodiment). Overall, these findings suggest that the B-ESEM-CU model of EES scores has adequate construct validity, with one exception: the positive association (both with the G-factor and some S-factors) with internalisation of appearance ideals was in the opposite direction to what we predicted (similar results were also obtained with the ESEM-CU model). Previously, Kling et al. [8] reported negative associations between embodiment and internalisation of appearance ideals, so this finding could be more carefully examined in future research.

Our findings also add to the literature in two other ways. First, across both split-half subsamples, we found that the ESEM models of the EES (i.e., the ESEM-CU model in the first split-half and the B-ESEM-CU model in the second split-half) evidenced complete measurement invariance across the two ethnic groups represented in our sample. This can be taken as additional evidence that the modelling of the EES in this manner is robust, at least in the national context of Cyprus. Second, and perhaps more importantly, across both split-half subsamples, we found no evidence of DIF; that is, participants of different ages and BMIs do not appear to respond differently to particular items in the EES. This is important because it suggests that the EES may be suitable for completion by all adult age and BMI groups and measures the same latent constructs irrespective of these respondent characteristics. Importantly, however, we did find that older participants and participants with lower BMIs had higher G-factor scores. While this is an area that requires further research, these findings are consistent with the suggestion that embodiment is a lived process that is likely to be affected by the physical state of one’s corporeal body [25].

### Limitations

The present findings should be considered in light of several limitations. First, in adopting the present analytic strategy, we sought to honour prior theoretical frameworks of the experience of embodiment [2–7] with empirical comprehensiveness. In adopting this strategy, however, we acknowledge that it is possible that an alternative factor structure may emerge as providing better fit to the data compared to the 6-factor B-ESEM-CU model. That is, our analytic strategy prioritises the theoretical underpinnings of the construct of experience of embodiment but does not necessarily allow us to test whether alternative models may explain the data better. We also cannot entirely rule out the possibility that our results are specific to the national context under investigation (i.e., Cyprus). However, given that previous work has not tested alternative models of the EES, as we have here, we suggest that it is incumbent on researchers to do so in previously examined national contexts.

Additionally, although the present study examined measurement invariance across participants who identified as Greek and Greek-Cypriot, we limited (for political and practical reasons) recruitment to the Republic of Cyprus (i.e., excluding respondents from Northern Cyprus, under Turkish occupation). Also, in terms of recruitment, our opportunistic method of recruitment means that our sample is unlikely to have been representative of the wider population of the Republic of Cyprus. We also did not collect additional information about our respondents, which may have been useful in terms of understanding experiences of embodiment, such as urbanicity and socioeconomic status [60]. As such, it would be useful in future research to recruit more representative samples of Cypriot adults, not to mention considering the extent to which the EES is invariant across factors such as socioeconomic status. Relatedly, it would also be useful to validate the Greek version of the EES in other Greek-speaking populations (e.g., in Greece). A final limitation is the fact that we did not assess test-retest reliability. Piran and colleagues [7] reported that EES scores were stable across a period of three weeks, but it would be useful to extend this by examining invariance of scores across time using the optimal model in the present study.

## Conclusion

The present results suggest that a B-ESEM-CU model provides an alternative way of modelling EES scores, at least for adults in the Republic of Cyprus, although we suggest that this model is also likely to prove robust in other national contexts for the reasons we have discussed above. We appreciate that this model may appear (more) complex compared to the model favoured by the scale developers (i.e., a HO-CFA model), but given the initial stages of the development of this instrument in a cross-national context, we suggest that there is a need to more carefully consider how best to model EES scores and the implications of EES modelling for both theory and practice. Indeed, we do not present the criticisms of the EES and its scoring above because we seek to stifle research on the experience of embodiment. Rather, we believe strongly that theoretical rigour should be supported by empirical data, and our data suggest that there may be alternative ways of modelling EES scores compared to the model presently favoured by researchers.

### Footnotes

^1^A power estimation was conducted using an α = .05, a Null RMSEA of .08, the sample size and models’ *df* and RMSEA values. Results revealed a power higher than 90% for both models.

^2^A power estimation was conducted and results revealed a power ranging from 80% to 100%.

^3^For comparison with previous studies, in S7 Table in S1 File, we also provide results taken from the ESEM-CU solution. Results showed significant correlations between all EES scales and other measures (except for BUA, ASC, RO and perfectionism).

## Supporting information

S1 Appendix Experience of Embodiment Scale in English and Greek. (DOCX)

S1 File Supplementary tables for EES factor analysis representations. (DOCX)

S2 File Data used in the present study. (CSV)

S3 File STROBE statement—checklist of items that should be included in reports of observational studies. (DOCX)
