# Supplementary material for: The Experience of Embodiment Scale: An examination of its psychometric properties in women from the Republic of Cyprus
Source: PLoS One. 2024 May 20;19(5):e0303268. doi: 10.1371/journal.pone.0303268 (PMC11104586; doi:10.1371/journal.pone.0303268)
Supplement: S1 Appendix — (DOCX) [file pone.0303268.s001.docx]

**Appendix 1.** Experience of Embodiment Scale *in English and Greek.*

| 1 | I feel in tune with my body / Νιώθω συντονισμένος/η με το σώμα μου. |
| --- | --- |
| 2 | I feel at one with my body / Νιώθω «ένα με το σώμα μου». |
| 3 | I feel “detached” and separate from my body / Αισθάνομαι «αποκομμένος/η» και χωρισμένος/η από το σώμα μου. |
| 4 | I feel depressed/anxious/scared in/about my body / Νιώθω θλιμμένος/η/αγχωμένος/η/φοβισμένος/η τόσο μέσα στο σώμα μου, όσο και για το σώμα μου. |
| 5 | I care more about how my body feels than about how it looks / Με νοιάζει περισσότερο το πώς αισθάνεται το σώμα μου παρά για το πώς φαίνεται. |
| 6 | I focus more on what my body can do than on its appearance / Εστιάζω περισσότερο στο τι μπορεί να κάνει το σώμα μου παρά στην εμφάνισή του. |
| 7 | My eating habits are a way for me to manage my emotions or how I have felt about myself / Οι διατροφικές μου συνήθειες είναι ένας τρόπος για μένα να διαχειριστώ τα συναισθήματά μου ή το πώς έχω νιώσει για τον εαυτό μου. |
| 8 | Generally I feel good/comfortable in my body / Γενικά αισθάνομαι καλά/άνετα μέσα στο σώμα μου. |
| 9 | I am proud of what my body can do / Είμαι περήφανος/η για το τι μπορεί να κάνει το σώμα μου. |
| 10 | I feel dissatisfied, envious and frustrated when I compare my body to others / Αισθάνομαι δυσαρέσκεια, ζήλια και εκνευρισμό όταν συγκρίνω το σώμα μου με άλλους. |
| 11 | I feel joy in my body / Νιώθω χαρά μέσα στο σώμα μου. |
| 12 | My body reduces my sense of self worth in the world / Το σώμα μου μειώνει την αίσθηση της αξίας του εαυτού μου στον κόσμο. |
| 13 | I sometimes tend to blame my body for difficulties I am having / Μερικές φορές τείνω να κατηγορώ το σώμα μου για δυσκολίες που αντιμετωπίζω. |
| 14 | I am comfortable with my sexual feelings/desires / Νιώθω άνετα με τα σεξουαλικά συναισθήματα/επιθυμίες μου. |
| 15 | I engage in potentially harmful or painful behaviours (e.g., disordered eating, bingeing, purging, denying physical needs, skin cutting, burning, drug use, excessive alcohol consumption) / Κάνω δυνητικά επιβλαβείς ή επώδυνες συμπεριφορές (π.χ. διαταραγμένη κατανάλωση φαγητού, υπερφαγία, εμετούς, δεν ακούω σωματικές μου ανάγκες, επιδερμικό κόψιμο, κάψιμο, χρήση ναρκωτικών, υπερβολική κατανάλωση αλκοόλ). |
| 16 | I have an eating disorder / Έχω διατροφική διαταραχή. |
| 17 | I take good care, and am respectful, of my body / Φροντίζω καλά και σέβομαι το σώμα μου. |
| 18 | I ignore the signs by body sends me (e.g., of hunger, stress, fatigue, illness/injury) / Αγνοώ τα σημάδια που μου στέλνει το σώμα μου (π.χ. πείνας, στρες, κόπωσης, ασθένειας/τραυματισμού). |
| 19 | I spend a lot of time/energy/money engaging in activities that I hope make me fit with cultural ideals of beauty (e.g., exercise, clothing, make-up, hair, plastic surgery, skin bleaching) / Ξοδεύω πολύ χρόνο/ενέργεια/χρήματα σε δραστηριότητες που ελπίζω να με κάνουν να μοιάζω με τα ιδανικά της κουλτούρας μου (π.χ. άσκηση, ρούχα, μακιγιάζ, μαλλιά, πλαστική χειρουργική, λεύκανση δέρματος). |
| 20 | I am comfortable voicing my views, opinions and beliefs / Νιώθω άνετα να εκφράσω την οπτική μου, τις απόψεις μου, και τις πεποιθήσεις μου. |
| 21 | I find it difficult to express my emotions / Το βρίσκω δύσκολο να εκφράσω τα συναισθήματά μου. |
| 22 | I am aware of my needs / Γνωρίζω τις ανάγκες μου. |
| 23 | It is hard for me to read/identify my feelings / Είναι δύσκολο για μένα να αναγνωρίσω/προσδιορίσω τα συναισθήματά μου. |
| 24 | I am comfortable with, and proud of, who I am / Είμαι άνετος/η και υπερήφανος/η για το ποιος είμαι. |
| 25 | I consider myself to be a powerful person / Θεωρώ τον εαυτό μου ως ένα ισχυρό άτομο. |
| 26 | I am aware of, and confident in, my strengths and abilities / Γνωρίζω και έχω αυτοπεποίθηση για τις δυνάμεις και τις ικανότητές μου. |
| 27 | My dissatisfaction with my body/appearance has a negative effect on my social life / Η δυσαρέσκεια μου με το σώμα / εμφάνιση μου έχει αρνητική επίδραση στην κοινωνική μου ζωή. |
| 28 | I feel disconnected from my own sense of sexual desire / Νιώθω αποσυνδεδεμένος/η από την αίσθηση της σεξουαλικής μου επιθυμίας. |
| 29 | I express what I want and need sexually / Εκφράζω τις σεξουαλικές μου επιθυμίες και ανάγκες. |
| 30 | I feel that I cannot express what I want or need in a dating/partnership relationship / Αισθάνομαι ότι δεν μπορώ να εκφράσω αυτό που θέλω ή χρειάζομαι σε μια ερωτική σχέση. |
| 31 | I have difficulty asserting myself with others in the world / Έχω δυσκολία να διεκδικήσω τα δικαιώματα μου με άλλους στον κόσμο. |
| 32 | I believe in my ability to accomplish what I desire in the world / Πιστεύω στην ικανότητά μου να πετύχω αυτό που επιθυμώ στον κόσμο. |
| 33 | I put a priority on listening to my body and its needs (e.g., stress, fatigue, hunger) / Δίνω προτεραιότητα στο να ακούω το σώμα μου και τις ανάγκες του (π.χ. άγχος, κόπωση, πείνα). |
| 34 | I constantly think about the way my body fits with cultural standards of beauty / Σκέφτομαι συνεχώς τον τρόπο που το σώμα μου ταιριάζει με τα πρότυπα ομορφιάς της κουλτούρας μου. |
