# Supplementary material for: The Experience of Embodiment Scale: An examination of its psychometric properties in women from the Republic of Cyprus
Source: PLoS One. 2024 May 20;19(5):e0303268. doi: 10.1371/journal.pone.0303268 (PMC11104586; doi:10.1371/journal.pone.0303268)
Supplement: S1 File — (DOCX) [file pone.0303268.s002.docx]

**Table 1.**

*Standardised Parameters Estimates from the Six-Factor Confirmatory Factor Analytic Representation with Correlated Uniqueness of the EES in the First Split-Half Subsample*

| Items | PBCC (λ) | BUA (λ) | AF (λ) | EESD (λ) | ASC (λ) | RO (λ) | δ |
| --- | --- | --- | --- | --- | --- | --- | --- |
| EES1 | **.887** |  |  |  |  |  | .213 |
| EES2 | **.900** |  |  |  |  |  | .189 |
| EES8 | **.919** |  |  |  |  |  | .155 |
| EES9 | **.701** |  |  |  |  |  | .509 |
| EES11 | **.906** |  |  |  |  |  | .180 |
| EES17 | **.812** |  |  |  |  |  | .341 |
| EES3 |  | **.803** |  |  |  |  | .355 |
| EES4 |  | **-.814** |  |  |  |  | .338 |
| EES7 |  | **.367** |  |  |  |  | .865 |
| EES10 |  | **.719** |  |  |  |  | .483 |
| EES12 |  | **.754** |  |  |  |  | .431 |
| EES13 |  | **.731** |  |  |  |  | .465 |
| EES27 |  | **.822** |  |  |  |  | .325 |
| EES34 |  | **.430** |  |  |  |  | .815 |
| EES20 |  |  | **.724** |  |  |  | .476 |
| EES21 |  |  | **.647** |  |  |  | .582 |
| EES24 |  |  | **.905** |  |  |  | .180 |
| EES25 |  |  | **.785** |  |  |  | .383 |
| EES26 |  |  | **.889** |  |  |  | .209 |
| EES31 |  |  | **.774** |  |  |  | .401 |
| EES32 |  |  | **.865** |  |  |  | .251 |
| EES14 |  |  |  | **.850** |  |  | .277 |
| EES28 |  |  |  | **.894** |  |  | .201 |
| EES29 |  |  |  | **.789** |  |  | .378 |
| EES30 |  |  |  | **.799** |  |  | .361 |
| EES15 |  |  |  |  | **.683** |  | .534 |
| EES16 |  |  |  |  | **.755** |  | .429 |
| EES18 |  |  |  |  | **.626** |  | .608 |
| EES22 |  |  |  |  | **.740** |  | .453 |
| EES23 |  |  |  |  | **.662** |  | .562 |
| EES33 |  |  |  |  | **.807** |  | .349 |
| EES5 |  |  |  |  |  | **.831** | .310 |
| EES6 |  |  |  |  |  | **.854** | .270 |
| EES19 |  |  |  |  |  | **.226** | .949 |
| ω | .943 | .879 | .926 | .901 | .862 | .705 |  |
| PBCC | - |  |  |  |  |  |  |
| BUA | .867 | - |  |  |  |  |  |
| AF | .599 | .620 | - |  |  |  |  |
| EESD | .653 | .673 | .658 | - |  |  |  |
| ASC | .720 | .737 | .665 | .667 | - |  |  |
| RO | .425 | .471 | .354 | .414 | .365 | - |  |

*Notes.* EES = Experience of Embodiment Scale; PBCC = Positive Body Connection and Comfort; λ = factor loadings; BUA = Body Unencumbered Adjustment; AF = Agency and Functionality; EESD = Experience and Expression of Sexual Desire; ASC = Attuned Self-Care; RO = Resisting Objectification; δ = Uniqueness; ω = McDonald’s omega. All correlations significant at *p* ≤ .01.

**Table 2.**

*Standardised Parameters Estimates from the Six-Factor Exploratory Structural Equation Modelling Representation with Correlated Uniqueness of the EES in the First Split-Half Subsample*

| Items | PBCC (λ) | BUA (λ) | AF (λ) | EESD (λ) | ASC (λ) | RO (λ) | δ |
| --- | --- | --- | --- | --- | --- | --- | --- |
| EES1 | **.815** | *.025* | .094 | -.091 | .098 | .179 | .131 |
| EES2 | **.799** | .067 | .163 | -.124 | .072 | .151 | .131 |
| EES8 | **.574** | .367 | *.015* | .255 | *-.032* | *-.012* | .142 |
| EES9 | **.553** | *.013* | .130 | .192 | *.027* | *-.054* | .474 |
| EES11 | **.559** | .306 | *.028* | .304 | *-.026* | *-.015* | .170 |
| EES17 | **.492** | .109 | *-.001* | .141 | .330 | *-.005* | .329 |
| EES3 | .532 | **.236** | .089 | -.105 | .183 | .126 | .363 |
| EES4 | -.370 | **-.438** | -.103 | *.018* | -.128 | *-.061* | .355 |
| EES7 | *-.019* | **.592** | -.102 | -.170 | .247 | *-.046* | .612 |
| EES10 | .131 | **.541** | .144 | *.081* | *-.003* | .077 | .450 |
| EES12 | .157 | **.517** | .087 | .157 | .067 | *.010* | .421 |
| EES13 | .237 | **.544** | *.049* | .089 | .079 | *-.059* | .420 |
| EES27 | *.032* | **.553** | .234 | .192 | *.055* | *.015* | .313 |
| EES34 | -.149 | **.562** | *.035* | *-.007* | *-.068* | .308 | .573 |
| EES20 | -.087 | *-.029* | **.493** | .122 | .236 | .146 | .479 |
| EES21 | -.228 | .087 | **.448** | *.032* | .445 | *.030* | .457 |
| EES24 | .117 | *.054* | **.679** | *.025* | .121 | .084 | .266 |
| EES25 | .059 | *-.025* | **.964** | *-.036* | -.205 | *-.011* | .197 |
| EES26 | .131 | *.024* | **.941** | *-.053* | -.104 | *-.030* | .140 |
| EES31 | -.144 | .179 | **.659** | .104 | .098 | *-.007* | .375 |
| EES32 | .125 | *.006* | **.839** | .089 | *-.047* | -.133 | .201 |
| EES14 | .199 | *-.009* | *-.010* | **.675** | *.033* | .115 | .323 |
| EES28 | .077 | .100 | *.054* | **.685** | *.048* | .064 | .284 |
| EES29 | *-.042* | -.112 | .082 | **.866** | *-.010* | .057 | .234 |
| EES30 | *-.033* | *.000* | *.055* | **.694** | .150 | *.021* | .363 |
| EES15 | *.090* | .333 | -.093 | *.026* | **.484** | *.068* | .475 |
| EES16 | .172 | .409 | -.110 | *.091* | **.404** | *-.037* | .421 |
| EES18 | *.016* | -.094 | -.104 | *.074* | **.942** | *-.057* | .185 |
| EES22 | *-.034* | *-.033* | .388 | .144 | **.337** | .097 | .492 |
| EES23 | -.193 | .088 | .320 | *-.036* | **.604** | .098 | .402 |
| EES33 | .257 | -.107 | .086 | .128 | **.615** | *-.026* | .339 |
| EES5 | *.022* | *-.011* | *-.035* | .094 | *-.011* | **.822** | .289 |
| EES6 | .128 | *-.059* | *-.048* | *.072* | *.000* | **.796** | .315 |
| EES19 | -.299 | .529 | *-.023* | *-.012* | -.133 | **.207** | .702 |
| ω | .913 | .819 | .923 | .876 | .832 | .718 |  |
| PBCC | - |  |  |  |  |  |  |
| BUA | .395 | - |  |  |  |  |  |
| AF | .363 | .288 | - |  |  |  |  |
| EESD | .375 | .400 | .558 | - |  |  |  |
| ASC | .366 | .366 | .395 | .432 | - |  |  |
| RO | .234 | .277 | .305 | .305 | .221 | - |  |

*Notes*. EES = Experience of Embodiment Scale; PBCC = Positive Body Connection and Comfort; λ = factor loadings; BUA = Body Unencumbered Adjustment; AF = Agency and Functionality; EESD = Experience and Expression of Sexual Desire; ASC = Attuned Self-Care; RO = Resisting Objectification; δ = Uniqueness; ω = McDonald’s omega; Non-significant cross-loadings and correlations are underlined and italicized.

**Table 3.**

*Standardised Parameters Estimates from the Six-Factor Confirmatory Factor Analytic Representation with Correlated Uniqueness of the EES in the Second Split-Half Subsample*

| Items | PBCC (λ) | BUA (λ) | AF (λ) | EESD (λ) | ASC (λ) | RO (λ) | δ |
| --- | --- | --- | --- | --- | --- | --- | --- |
| EES1 | **.846** |  |  |  |  |  | .285 |
| EES2 | **.888** |  |  |  |  |  | .212 |
| EES8 | **.890** |  |  |  |  |  | .208 |
| EES9 | **.720** |  |  |  |  |  | .482 |
| EES11 | **.881** |  |  |  |  |  | .224 |
| EES17 | **.815** |  |  |  |  |  | .335 |
| EES3 |  | **.825** |  |  |  |  | .319 |
| EES4 |  | **-.839** |  |  |  |  | .296 |
| EES7 |  | **.410** |  |  |  |  | .832 |
| EES10 |  | **.722** |  |  |  |  | .478 |
| EES12 |  | **.845** |  |  |  |  | .286 |
| EES13 |  | **.695** |  |  |  |  | .517 |
| EES27 |  | **.826** |  |  |  |  | .317 |
| EES34 |  | **.428** |  |  |  |  | .817 |
| EES20 |  |  | **.643** |  |  |  | .587 |
| EES21 |  |  | **.668** |  |  |  | .554 |
| EES24 |  |  | **.919** |  |  |  | .155 |
| EES25 |  |  | **.789** |  |  |  | .377 |
| EES26 |  |  | **.808** |  |  |  | .347 |
| EES31 |  |  | **.746** |  |  |  | .443 |
| EES32 |  |  | **.775** |  |  |  | .400 |
| EES14 |  |  |  | **.847** |  |  | .283 |
| EES28 |  |  |  | **.845** |  |  | .286 |
| EES29 |  |  |  | **.829** |  |  | .313 |
| EES30 |  |  |  | **.815** |  |  | .336 |
| EES15 |  |  |  |  | **.785** |  | .384 |
| EES16 |  |  |  |  | **.748** |  | .441 |
| EES18 |  |  |  |  | **.654** |  | .572 |
| EES22 |  |  |  |  | **.713** |  | .492 |
| EES23 |  |  |  |  | **.606** |  | .633 |
| EES33 |  |  |  |  | **.736** |  | .459 |
| EES5 |  |  |  |  |  | **.710** | .496 |
| EES6 |  |  |  |  |  | **.939** | .118 |
| EES19 |  |  |  |  |  | **.308** | .905 |
| ω | .936 | .890 | .909 | .901 | .858 | .716 |  |
| PBCC | - |  |  |  |  |  |  |
| BUA | .852 | - |  |  |  |  |  |
| AF | .586 | .615 | - |  |  |  |  |
| EESD | .576 | .618 | .601 | - |  |  |  |
| ASC | .758 | .775 | .675 | .576 | - |  |  |
| RO | .379 | .445 | .396 | .219 | .381 | - |  |

*Notes*. EES = Experience of Embodiment Scale; PBCC = Positive Body Connection and Comfort; λ = factor loadings; BUA = Body Unencumbered Adjustment; AF = Agency and Functionality; EESD = Experience and Expression of Sexual Desire; ASC = Attuned Self-Care; RO = Resisting Objectification; δ = Uniqueness; ω = McDonald’s omega. All correlations significant at *p* ≤ .01.

**Table 4.**

*Standardised Parameters Estimates from the Hierarchical Order Confirmatory Factor Analytic Representation with Correlated Uniqueness of the EES in the Second Split-Half Subsample*

| Items |  | PBCC (λ) | BUA (λ) | AF (λ) | EESD (λ) | ASC (λ) | RO (λ) | δ |
| --- | --- | --- | --- | --- | --- | --- | --- | --- |
| EES1 |  | **.845** |  |  |  |  |  | .285 |
| EES2 |  | **.887** |  |  |  |  |  | .213 |
| EES8 |  | **.892** |  |  |  |  |  | .205 |
| EES9 |  | **.718** |  |  |  |  |  | .484 |
| EES11 |  | **.882** |  |  |  |  |  | .222 |
| EES17 |  | **.814** |  |  |  |  |  | .338 |
| EES3 |  |  | **.827** |  |  |  |  | .316 |
| EES4 |  |  | **-.840** |  |  |  |  | .294 |
| EES7 |  |  | **.408** |  |  |  |  | .834 |
| EES10 |  |  | **.721** |  |  |  |  | .480 |
| EES12 |  |  | **.846** |  |  |  |  | .285 |
| EES13 |  |  | **.693** |  |  |  |  | .520 |
| EES27 |  |  | **.825** |  |  |  |  | .320 |
| EES34 |  |  | **.427** |  |  |  |  | .818 |
| EES20 |  |  |  | **.646** |  |  |  | .583 |
| EES21 |  |  |  | **.657** |  |  |  | .568 |
| EES24 |  |  |  | **.923** |  |  |  | .148 |
| EES25 |  |  |  | **.790** |  |  |  | .375 |
| EES26 |  |  |  | **.811** |  |  |  | .342 |
| EES31 |  |  |  | **.735** |  |  |  | .460 |
| EES32 |  |  |  | **.776** |  |  |  | .397 |
| EES14 |  |  |  |  | **.848** |  |  | .282 |
| EES28 |  |  |  |  | **.845** |  |  | .287 |
| EES29 |  |  |  |  | **.828** |  |  | .314 |
| EES30 |  |  |  |  | **.814** |  |  | .337 |
| EES15 |  |  |  |  |  | **.785** |  | .384 |
| EES16 |  |  |  |  |  | **.748** |  | .441 |
| EES18 |  |  |  |  |  | **.655** |  | .571 |
| EES22 |  |  |  |  |  | **.713** |  | .492 |
| EES23 |  |  |  |  |  | **.605** |  | .634 |
| EES33 |  |  |  |  |  | **.736** |  | .458 |
| EES5 |  |  |  |  |  |  | **.710** | .496 |
| EES6 |  |  |  |  |  |  | **.939** | .118 |
| EES19 |  |  |  |  |  |  | **.306** | .906 |
| ω |  | .936 | .890 | .908 | .901 | .858 | .715 |  |
|  |  | PBCC | BUA | AF | EESD | ASC | RO | ω |
| 2^nd^ order factor | γ | .883 | .924 | .716 | .691 | .863 | .457 | .895 |
|  | ζ | .220 | .146 | .487 | .523 | .255 | .791 |  |

*Notes*. EES = Experience of Embodiment Scale; PBCC = Positive Body Connection and Comfort; λ = factor loadings; BUA = Body Unencumbered Adjustment; AF = Agency and Functionality; EESD = Experience and Expression of Sexual Desire; ASC = Attuned Self-Care; RO = Resisting Objectification; δ = Uniqueness; ω = McDonald’s omega; γ = 2^nd^ order factor loading; ζ = 2^nd^ order measurement error.

**Table 5.**

*Standardised Parameters Estimates from the Bifactor Confirmatory Factor Analytic Representation with Correlated Uniqueness of the EES in the Second Split-Half Subsample*

| Items | PBCC (λ) S-factor | BUA (λ) S-factor | AF (λ) S-factor | EESD (λ) S-factor | ASC (λ) S-factor | RO (λ) S-factor | G-factor | δ |
| --- | --- | --- | --- | --- | --- | --- | --- | --- |
| EES1 | **.651** |  |  |  |  |  | **.667** | .131 |
| EES2 | **.587** |  |  |  |  |  | **.722** | .134 |
| EES8 | **.150** |  |  |  |  |  | **.852** | .252 |
| EES9 | **.107** |  |  |  |  |  | **.681** | .524 |
| EES11 | **.193** |  |  |  |  |  | **.836** | .263 |
| EES17 | **.067** |  |  |  |  |  | **.777** | .391 |
| EES3 |  | **.008** |  |  |  |  | **.802** | .356 |
| EES4 |  | **-.231** |  |  |  |  | **-.782** | .335 |
| EES7 |  | **.334** |  |  |  |  | **.353** | .764 |
| EES10 |  | **.469** |  |  |  |  | **.632** | .380 |
| EES12 |  | **.471** |  |  |  |  | **.754** | .210 |
| EES13 |  | **.379** |  |  |  |  | **.618** | .474 |
| EES27 |  | **.245** |  |  |  |  | **.766** | .353 |
| EES34 |  | **.347** |  |  |  |  | **.364** | .747 |
| EES20 |  |  | **.435** |  |  |  | **.469** | .591 |
| EES21 |  |  | **.377** |  |  |  | **.495** | .613 |
| EES24 |  |  | **.489** |  |  |  | **.707** | .261 |
| EES25 |  |  | **.726** |  |  |  | **.494** | .229 |
| EES26 |  |  | **.728** |  |  |  | **.516** | .204 |
| EES31 |  |  | **.428** |  |  |  | **.553** | .511 |
| EES32 |  |  | **.512** |  |  |  | **.566** | .417 |
| EES14 |  |  |  | **.529** |  |  | **.610** | .348 |
| EES28 |  |  |  | **.565** |  |  | **.588** | .335 |
| EES29 |  |  |  | **.746** |  |  | **.536** | .157 |
| EES30 |  |  |  | **.541** |  |  | **.565** | .388 |
| EES15 |  |  |  |  | **.057** |  | **.692** | .518 |
| EES16 |  |  |  |  | **.043** |  | **.661** | .561 |
| EES18 |  |  |  |  | **.834** |  | **.538** | .014 |
| EES22 |  |  |  |  | **.268** |  | **.616** | .549 |
| EES23 |  |  |  |  | **.813** |  | **.493** | .096 |
| EES33 |  |  |  |  | **.298** |  | **.633** | .511 |
| EES5 |  |  |  |  |  | **.710** | **.322** | .392 |
| EES6 |  |  |  |  |  | **.754** | **.428** | .248 |
| EES19 |  |  |  |  |  | **.234** | **.148** | .923 |
| ω | .645 | .630 | .829 | .822 | .704 | .648 | .969 |  |

*Notes*. EES = Experience of Embodiment Scale; PBCC = Positive Body Connection and Comfort; λ = factor loadings; S-factor = specific factor; BUA = Body Unencumbered Adjustment; AF = Agency and Functionality; EESD = Experience and Expression of Sexual Desire; ASC = Attuned Self-Care; RO = Resisting Objectification; G-factor = global factor; δ = Uniqueness; ω = McDonald’s omega.

**Table 6.**

*Standardised Parameters Estimates from the Six-Factor Exploratory Structural Equation Modeling Representation with Correlated Uniqueness of the EES in the Second Split-Half Subsample*

| Items | PBCC (λ) | BUA (λ) | AF (λ) | EESD (λ) | ASC (λ) | RO (λ) | δ |
| --- | --- | --- | --- | --- | --- | --- | --- |
| EES1 | **.811** | *.021* | .157 | -.119 | .131 | -.130 | .187 |
| EES2 | **.850** | *.057* | .195 | -.103 | .070 | -.139 | .103 |
| EES8 | **.549** | .385 | -.061 | .128 | *.000* | .174 | .193 |
| EES9 | **.489** | *.080* | *.062* | .265 | *-.074* | .232 | .441 |
| EES11 | **.596** | .270 | *-.045* | .118 | *.034* | .226 | .201 |
| EES17 | **.437** | *.032* | -.114 | .200 | .406 | .099 | .313 |
| EES3 | .489 | **.346** | .111 | *.001* | .139 | -.170 | .314 |
| EES4 | -.256 | **-.563** | *-.042* | -.066 | -.148 | .090 | .294 |
| EES7 | *-.067* | **.509** | -.134 | *.002* | .206 | -.100 | .687 |
| EES10 | .141 | **.632** | *.058* | *.013* | *-.058* | .192 | .389 |
| EES12 | .151 | **.718** | .129 | *-.009* | *.010* | .080 | .228 |
| EES13 | .139 | **.661** | *.026* | .074 | *-.021* | -.069 | .438 |
| EES27 | *.039* | **.528** | .122 | .266 | .084 | *.056* | .321 |
| EES34 | -.183 | **.490** | .114 | *-.071* | *.046* | .258 | .638 |
| EES20 | *-.080* | *.082* | **.523** | .169 | *.056* | *.026* | .582 |
| EES21 | -.177 | *.073* | **.460** | .146 | .301 | *.009* | .518 |
| EES24 | .105 | .148 | **.651** | *.009* | .150 | *.048* | .254 |
| EES25 | *.045* | *.046* | **.848** | *-.033* | *-.049* | .071 | .249 |
| EES26 | .162 | *-.031* | **.894** | *-.037* | -.068 | *.002* | .173 |
| EES31 | *-.053* | .112 | **.497** | .282 | *.052* | *.050* | .470 |
| EES32 | .105 | *-.048* | **.571** | .193 | .081 | *.067* | .424 |
| EES14 | .127 | *.070* | .152 | **.655** | *-.044* | *.002* | .367 |
| EES28 | *.000* | .091 | *.008* | **.733** | .149 | -.075 | .282 |
| EES29 | *.002* | -.073 | .142 | **.893** | -.047 | *-.015* | .178 |
| EES30 | *.001* | *.069* | .123 | **.682** | *.073* | -.073 | .367 |
| EES15 | *.043* | .358 | -.084 | *.066* | **.530** | *-.011* | .361 |
| EES16 | .095 | .317 | -.104 | *.048* | **.515** | *-.034* | .419 |
| EES18 | *.058* | -.120 | -.125 | *.069* | **.839** | .083 | .339 |
| EES22 | *.013* | *-.040* | .451 | -.082 | **.496** | *.010* | .441 |
| EES23 | -.172 | *.046* | .307 | *-.076* | **.630** | *-.016* | .476 |
| EES33 | .277 | -.213 | *-.035* | .074 | **.682** | .130 | .370 |
| EES5 | .099 | *-.027* | *-.002* | *-.051* | *.053* | **.792** | .351 |
| EES6 | .128 | *-.040* | .165 | -.075 | .074 | **.737** | .332 |
| EES19 | -.275 | .388 | *.053* | -.184 | *.101* | **.197** | .776 |
| ω | .906 | .857 | .881 | .880 | .850 | .671 |  |
| PBCC | - |  |  |  |  |  |  |
| BUA | .460 | - |  |  |  |  |  |
| AF | .342 | .293 | - |  |  |  |  |
| EESD | .359 | .385 | .371 | - |  |  |  |
| ASC | .456 | .515 | .407 | .448 | - |  |  |
| RO | .133 | .291 | .225 | .149 | .177 | - |  |

Notes. EES = Experience of Embodiment Scale; PBCC = Positive Body Connection and Comfort; λ = factor loadings; BUA = Body Unencumbered Adjustment; AF = Agency and Functionality; EESD = Experience and Expression of Sexual Desire; ASC = Attuned Self-Care; RO = Resisting Objectification; δ = Uniqueness; ω = McDonald’s omega; Non-significant cross-loadings and correlations are underlined and italicized.

**Table 7.**

*Standardised Parameters Estimates from the Hierarchical Order Exploratory Structural Equation Modeling Representation with Correlated Uniqueness of the EES in the Second Split-Half Subsample*

| Items |  | PBCC (λ) | BUA (λ) | AF (λ) | EESD (λ) | ASC (λ) | RO (λ) | δ |
| --- | --- | --- | --- | --- | --- | --- | --- | --- |
| EES1 |  | **.815** | *.036* | .151 | -.121 | .131 | -.148 | .186 |
| EES2 |  | **.857** | .058 | .197 | -.104 | .069 | -.143 | .103 |
| EES8 |  | **.549** | .445 | *-.050* | .099 | *-.023* | .159 | .192 |
| EES9 |  | **.479** | .146 | .095 | .248 | -.118 | .201 | .446 |
| EES11 |  | **.591** | .338 | *-.027* | .092 | *.001* | .204 | .203 |
| EES17 |  | **.430** | .095 | -.089 | .177 | .375 | *.046* | .326 |
| EES3 |  | .505 | **.331** | .087 | *-.004* | .165 | -.159 | .317 |
| EES4 |  | -.272 | **-.546** | *-.013* | *-.054* | -.182 | *.064* | .298 |
| EES7 |  | *-.056* | **.498** | -.162 | *-.013* | .237 | *-.079* | .683 |
| EES10 |  | .149 | **.634** | *.038* | *-.002* | *-.041* | .234 | .388 |
| EES12 |  | .152 | **.731** | .130 | -.009 | .010 | .082 | .217 |
| EES13 |  | .152 | **.645** | *-.004* | *.062* | *.015* | *-.034* | .440 |
| EES27 |  | *.050* | **.512** | .107 | .259 | *.106* | .084 | .322 |
| EES34 |  | -.173 | **.466** | *.089* | *-.076* | *.072* | .313 | .636 |
| EES20 |  | *-.074* | *.025* | **.514** | .199 | *.074* | *.064* | .583 |
| EES21 |  | -.170 | *.008* | **.445** | .179 | .328 | *.042* | .516 |
| EES24 |  | .104 | .114 | **.641** | *.035* | .163 | .076 | .255 |
| EES25 |  | *.035* | *.015* | **.844** | *.003* | *-.045* | .105 | .248 |
| EES26 |  | .163 | -.032 | **.901** | -.037 | -.068 | .002 | .162 |
| EES31 |  | *-.050* | *.074* | **.498** | .311 | *.055* | *.076* | .467 |
| EES32 |  | .099 | *-.055* | **.585** | .216 | *.067* | *.067* | .423 |
| EES14 |  | .134 | *.056* | .165 | **.660** | *-.045* | *.001* | .365 |
| EES28 |  | *.009* | *.071* | *.021* | **.731** | .159 | *-.086* | .282 |
| EES29 |  | .002 | -.074 | .143 | **.900** | -.046 | -.015 | .183 |
| EES30 |  | *.009* | *.042* | .133 | **.687** | *.083* | *-.073* | .366 |
| EES15 |  | *.054* | .355 | -.102 | *.050* | **.550** | *-.012* | .360 |
| EES16 |  | .105 | .321 | -.118 | *.031* | **.530** | *-.041* | .418 |
| EES18 |  | .058 | -.123 | -.126 | .070 | **.832** | .085 | .330 |
| EES22 |  | .018 | *-.085* | .439 | *-.053* | **.511** | *.020* | .439 |
| EES23 |  | *-.164* | *-.013* | .281 | *-.048* | **.666** | *.001* | .470 |
| EES33 |  | .272 | -.166 | *-.010* | *.067* | **.645** | *.071* | .389 |
| EES5 |  | .100 | -.028 | -.002 | -.051 | .053 | **.815** | .299 |
| EES6 |  | .115 | *.016* | .201 | *-.079* | *.023* | **.717** | .352 |
| EES19 |  | -.267 | .350 | *.017* | -.187 | *.146* | **.252** | .775 |
| ω |  | .905 | .852 | .881 | .881 | .853 | .691 |  |
|  |  | PBCC | BUA | AF | EESD | ASC | RO | ω |
| 2nd order factor | γ | .617 | .651 | .532 | .596 | .761 | .293 | .754 |
|  | ζ | .619 | .576 | .717 | .645 | .420 | .914 |  |

*Notes.* EES = Experience of Embodiment Scale; PBCC = Positive Body Connection and Comfort; λ = factor loadings; BUA = Body Unencumbered Adjustment; AF = Agency and Functionality; EESD = Experience and Expression of Sexual Desire; ASC = Attuned Self-Care; RO = Resisting Objectification; δ = Uniqueness; ω = McDonald’s omega; γ = 2^nd^ order factor loading; ζ = 2^nd^ order measurement error; Non-significant cross-loadings are underlined and italicised.

**Table 8.**

*Standardised Parameters Estimates from the Bifactor Exploratory Structural Equation Modelling Representation with Correlated Uniqueness of the EES in the Second Split-Half Subsample*

| Items | PBCC (λ) S-factor | BUA (λ) S-factor | AF (λ) S-factor | EESD (λ) S-factor | ASC (λ) S-factor | RO (λ) S-factor | G-factor | δ |
| --- | --- | --- | --- | --- | --- | --- | --- | --- |
| EES1 | **.596** | *-.012* | *.003* | *-.046* | .091 | -.056 | **.662** | .193 |
| EES2 | **.674** | *.012* | *.033* | *-.011* | *.045* | -.044 | **.691** | .063 |
| EES8 | **.141** | .133 | -.108 | -.087 | -.184 | *.034* | **.853** | .180 |
| EES9 | ***.077*** | -.092 | *.023* | *.040* | -.235 | .080 | **.712** | .415 |
| EES11 | **.179** | *.053* | -.096 | -.083 | -.157 | .086 | **.849** | .196 |
| EES17 | ***-.024*** | -.168 | -.126 | -.064 | *.060* | -.115 | **.843** | .224 |
| EES3 | .373 | **.221** | *-.011* | *.029* | .096 | -.108 | **.703** | .297 |
| EES4 | -.128 | **-.351** | *.046* | *-.017* | -.069 | .083 | **-.746** | .290 |
| EES7 | -.169 | **.319** | -.144 | *-.079* | *.094* | -.139 | **.391** | .662 |
| EES10 | *.030* | **.417** | *-.019* | *-.029* | -.083 | .200 | **.629** | .382 |
| EES12 | *-.004* | **.446** | *.044* | -.086 | *-.053* | .061 | **.748** | .225 |
| EES13 | *-.004* | **.404** | *-.038* | *-.017* | *-.072* | -.093 | **.624** | .432 |
| EES27 | -.091 | **.314** | .064 | .151 | *.008* | *.026* | **.740** | .318 |
| EES34 | *-.069* | **.403** | *.063* | *.006* | .076 | .324 | **.321** | .615 |
| EES20 | *-.005* | *.060* | **.448** | .181 | .076 | *.061* | **.416** | .580 |
| EES21 | *-.015* | .110 | **.402** | .233 | .292 | .085 | **.437** | .488 |
| EES24 | .063 | .064 | **.528** | *.000* | .115 | *.045* | **.670** | .250 |
| EES25 | *-.025* | *-.020* | **.720** | -.068 | *-.030* | *.042* | **.505** | .219 |
| EES26 | .118 | -.074 | **.738** | *-.038* | *-.025* | *-.007* | **.513** | .171 |
| EES31 | -.114 | *.024* | **.419** | .195 | *.013* | *.020* | **.553** | .466 |
| EES32 | -.103 | -.140 | **.488** | .065 | *-.012* | *-.031* | **.599** | .367 |
| EES14 | .075 | *.019* | .108 | **.533** | *-.040* | *.020* | **.589** | .349 |
| EES28 | -.071 | *.036* | *.000* | **.586** | .113 | -.074 | **.606** | .264 |
| EES29 | -.059 | -.103 | .133 | **.704** | -.053 | *-.037* | **.540** | .177 |
| EES30 | *-.024* | *.038* | .107 | **.568** | .082 | *-.053* | **.553** | .349 |
| EES15 | -.115 | .187 | -.110 | *-.031* | **.267** | -.079 | **.702** | .368 |
| EES16 | -.104 | .137 | -.121 | -.082 | **.218** | -.130 | **.683** | .419 |
| EES18 | -.132 | -.146 | -.113 | *-.025* | **.455** | *-.019* | **.615** | .363 |
| EES22 | .183 | *.027* | .362 | *.061* | **.433** | .095 | **.489** | .395 |
| EES23 | .089 | .159 | .256 | .153 | **.539** | .110 | **.400** | .415 |
| EES33 | *-.008* | -.243 | *-.043* | *-.046* | **.369** | *.010* | **.677** | .342 |
| EES5 | -.059 | *-.022* | *-.003* | -.080 | *-.045* | **.729** | **.341** | .340 |
| EES6 | *.013* | *-.020* | .138 | -.068 | *-.004* | **.706** | **.411** | .308 |
| EES19 | *.000* | .424 | *.025* | *.051* | .241 | **.340** | ***.053*** | .640 |
| ω | .692 | .720 | .846 | .834 | .693 | .710 | .971 |  |

*Notes*. EES = Experience of Embodiment Scale; PBCC = Positive Body Connection and Comfort; λ = factor loadings; S-factor = specific factor; BUA = Body Unencumbered Adjustment; AF = Agency and Functionality; EESD = Experience and Expression of Sexual Desire; ASC = Attuned Self-Care; RO = Resisting Objectification; G-factor = global factor; δ = Uniqueness; ω = McDonald’s omega; Non-significant cross-loadings are underlined and italicised.

**Table 9.**

*Construct Validity Analyses from the Six-Factor Exploratory Structural Equation Modeling Representation with Correlated Uniqueness of the EES in the Overall Sample*

|  | PBCC | BUA | AF | EESD | ASC | RO |
| --- | --- | --- | --- | --- | --- | --- |
| Body appreciation | .710** | .651** | .568** | .627** | .587** | .354** |
| Eating restriction | -.221** | -.470** | -.071* | -.145** | -.250** | -.270** |
| Internalisation of appearance ideals | .244** | .479** | .306** | .274** | .253** | .568** |
| Life satisfaction | .394** | .322** | .492** | .398** | .385** | .132** |
| Perfectionism | .186** | -.019 | .385** | .197** | .024 | -.030 |
| Self-esteem | .465** | .499** | .751** | .511** | .522** | .253** |

*Notes*. EES = Experience of Embodiment Scale; PBCC = Positive Body Connection and Comfort; BUA = Body Unencumbered Adjustment; AF = Agency and Functionality; EESD = Experience and Expression of Sexual Desire; ASC = Attuned Self-Care; RO = Resisting Objectification. * p ≤ .05; ** p ≤ .01.
